# Correlates of children’s time-specific physical activity: A review of the literature

**DOI:** 10.1186/1479-5868-9-50

**Published:** 2012-04-30

**Authors:** Rebecca M Stanley, Kate Ridley, James Dollman

**Affiliations:** 1 Health and Use of Time Group, School of Health Sciences, University of South Australia, GPO Box 2471, Adelaide, South Australia, 5001, Australia; 2 School of Education, Flinders University, GPO Box 2100, Adelaide, South Australia, 5001, Australia

**Keywords:** Ecological model, Children, Adolescents, Preadolescents, Public health, Physical activity

## Abstract

Assessment of correlates of physical activity occurring at different times of the day, locations and contexts, is imperative to understanding children’s physical activity behaviour. The purpose of this review was to identify the correlates of children’s physical activity (aged 8–14 years) occurring during the school break time and after-school periods. A review was conducted of the peer-reviewed literature, published between 1990 and January 2011. A total of 22 studies (12 school break time studies, 10 after-school studies) were included in the review. Across the 22 studies, 17 studies were cross-sectional and five studies were interventions. In the school break time studies, 39 potential correlates were identified, of which gender and age were consistently associated with school break time physical activity in two or more studies, and family affluence, access to a gym, access to four or more physical activity programs and the condition of a playing field were all associated with school break time physical activity in only one study. Access to loose and fixed equipment, playground markings, size of and access to play space and the length of school break time were all positively associated with changes in school break time physical activity in intervention studies. Thirty-six potential correlates of after-school physical activity were identified. Gender (with boys more active), younger age, lower body mass index (for females), lower TV viewing/playing video games, and greater access to facilities were associated with higher levels of after-school physical activity in two or more studies. Parent supervision was negatively associated with females’ after-school physical activity in one study. This review has revealed a relatively small number of studies investigating the school break time and after-school periods in the specified age range and only a few correlates have demonstrated a consistent association with physical activity. This highlights the infancy of this area and a need for further investigation into time-specific physical activity behaviour so that interventions designed for these specific periods can target the important correlates.

## Background

Physical activity (PA) behaviour is influenced by a complex interaction of factors in different domains, including intrapersonal (e.g. beliefs, attitudes and efficacy), social (e.g. peer, teacher and parental support) and physical environment (e.g. geographical location and topography) [1]. There is evidence that PA is declining in specific contexts, such as active transport, organised sports, leisure time PA and physical education [2,3]. With knowledge of the health benefits of regular PA [4-6], many interventions have attempted to increase children’s PA levels in specific contexts with varied levels of success [7-9]. To improve the effectiveness of interventions it may be necessary to understand the key factors that influence PA behaviour in different contexts, such as location and time-specific contexts.

In 2005, Giles-Corti et al. [10] called for increased specificity in correlate research that utilises an ecological framework. Motivation for this statement [10] was prompted by the modest performance of PA interventions [8] and explanation of small amounts of variance in PA behaviour by behavioural models [11,12]. Two recent systematic reviews on the effectiveness of PA interventions in youth found 38% [7] and 47% [8] had a statistically significant, positive effect on PA, but the effects were small and short lived. These modest outcomes may be in part due to an inadequate understanding of the primary correlates that impact PA behaviour for a particular population in a specific context. Cross-sectional studies guided by uni-dimensional behavioural theories and models [12] tend to explain small amounts of variance of PA and do not capture the complex dimensions of such a multi-level behaviour [11,13]. Ajzen and Fishbein’s theory of reasoned action and theory of planned behaviour [14] are commonly used theories in PA studies which tend to concentrate on intrapersonal factors, with limited recognition of external influences, such as the physical environment. Spence and Lee [15] found that these models only explain 20% to 40% of variance in PA behaviour in children and adolescents, leaving a large percentage of the variance in PA unexplained. This may be due to the difficulties in measuring PA and the associated correlates but also demonstrates the complexity of PA behaviour [16,17] and the lack of specificity of behavioural theories and models [12]. Researchers have acknowledged the limitations of these theoretical approaches and research is now shifting towards the development and use of ecological models, which posit that behaviour is multidimensional and is influenced by an interaction of factors across intrapersonal, sociocultural and physical environment domains [10,15,18].

The body of literature describing PA correlates in children, adolescents and adults is extensive and has substantially escalated in recent years [10]. A number of high quality reviews have attempted to synthesise the current literature for understanding habitual PA [11,19-24], with very few focusing on correlates of PA in specific contexts [25]. The diversity in research design, theoretical approaches, measurement approaches, analytical approaches, population groups, correlates investigated and PA outcomes across the literature makes it difficult to understand the evidence and to draw appropriate conclusions [11]. Previous reviews have used habitual PA, an overall measure of total PA, as the dependent variable without consideration of the specific type of behaviour (e.g. walking, moderate to vigorous PA), location (e.g. home, neighbourhood, school) or time of day in which PA is performed [11,19-24]. Furthermore, these reviews tend to combine conceptually similar correlates. For example, access to specific spaces, such as green spaces and bitumen spaces, are collapsed into one ‘access to space’ correlate. While this is done for ease of interpretation, the level of contextual information is lost, which may be crucial in understanding PA behaviour in specific contexts. Ferreira et al. [25] did conduct an important review of environmental correlates using PA performed in the home, school and neighbourhood context as the dependent variable. However, this review did not include the psychological or behavioural domains of the ecological model, exposing an important gap in the review literature. While these reviews are useful for obtaining an overview of correlates that influence children’s habitual PA, applying these findings to understand the influences on specific PA behaviours in different contexts may be less useful as the correlates may not be applicable to the specific context under investigation. Similarly, when using these correlates in intervention design there is no assurance that the dominant correlates will be captured or targeted in context-specific interventions, thus reducing the ability to effectively influence PA behaviour [11,26].

Ommundsen et al. [27] conducted a study in Norway as part of the European Youth Heart Study to demonstrate that psycho-social and perceived environmental predictors of PA are location-specific. They concluded that there were some similarities but also some important differences in PA predictors dependent upon age, gender and location. For example, peer support, enjoyment and perceived competence were significant predictors of before, during and after-school activities, whereas parental support only predicted after-school activity and teacher support only predicted free play during school. Spink et al. [28] investigated the predictors of unstructured and structured activity in active youth and found that enjoyment, perceived competence, parental support and coaches’ support were associated with structured activity, while friends’ participation was associated with unstructured activity. This emerging evidence of context-specific correlates signifies a need to alter the way we review the current correlate literature, which can be used to inform the refinement and increase the specificity of ecological models.

The school break time (i.e. lunchtime and morning/afternoon breaks) and weekday after-school periods are two crucial times of a day where children generally have discretion over the activities in which they engage [29]. These two periods of the day are examples of time-specific contexts. Research has shown that children can obtain up to one-third of their recommended daily moderate-to-vigorous PA during the school break time period [30] and up to half of their daily recommended PA in the after-school period [31,32]. The importance of the school break time and after-school periods for PA promotion has prompted the need to review the correlates that influence children’s engagement in school break time and after-school PA. The purpose of this systematic review is to identify the correlates of children’s school break time and after-school PA. This research will build on recent correlate reviews by looking specifically at the school break time and after-school periods and identifying the contextual information that tends to be missing from the current literature.

## Methods

### Search strategy

A systematic search of the literature was conducted to identify studies that assessed potential correlates of school break time and after-school PA. Peer-reviewed journal articles (in the English language), published between 1990 and January 2011 were searched using electronic databases (Medline, Scopus, EbscoHost [Academic Search Premier, CINAHL, ERIC, Health Source: Nursing/Academic Edition, Pre-CINAHL, PsycARTICLES, Psychology and Behavioral Sciences Collection, PsycINFO, SPORTDiscus, Academic Search Alumni Edition] and Web of Knowledge), as well as conducting manual searches of reference lists of retrieved studies. The following keyword combinations were used: children, adolescents, youth, younger person, young people, physical activity, sport, exercise, free play, play, leisure activity, organised activity, non-organised activity, transport, active transport, active commuting, recreation, correlate, determinant, predictor, factor, association, influencers, recess, lunchtime, lunch break, recess break, recess time, school break, after-school, and environment, physical environment, facilities, school, built environment, psychosocial, social environment, sociocultural environment, neighbourhood, perceived environment. The truncation symbol was used to ensure all terms with the respective prefix were identified. The search strategy (databases and search terms) was validated by an experienced research librarian. Titles and abstracts of potential articles were reviewed for relevance. The full-text copy of the article was retrieved for all abstracts fitting the selection criteria.

### Selection criteria

#### Types of articles

Studies that reported cross-sectional, longitudinal associations or experimental results were included in the review. Questionnaire validation studies focusing on testing psychometric properties of measurement tools were only included if they explored the association between a correlate and time-specific PA. Qualitative studies, expert opinion, conference proceedings, dissertations and case studies were excluded.

#### Sample

Studies were required to have been conducted with participants of preadolescent years in primary schools. There is some confusion as to how to define preadolescence by age in years. Typically, preadolescence is defined as 9–13 years [33] but some reports include children aged 8–12 years, 9–14 years, 9–13 years or 12–14 years [34,35]. For this review, preadolescence was defined as 8–14 years to capture all potential studies focusing on this age group. Studies have shown that PA levels decline as children reach puberty and preadolescence and continue to decline rapidly through adolescence [36]. These observations highlight this age group as an important target for disease prevention and establishment of lifelong healthy behaviours, particularly in relation to obesity and cardiovascular risk factors [37,38]. Due to variability of the age at which children transition from primary to secondary school across countries and likely differences between social and environmental characteristics of these two school settings [39,40], only preadolescent studies containing primary school children’s data were included. If an identified study included students from both primary and secondary schools and conducted separate age or grade analyses, only the findings corresponding to primary level were used.

Only studies for healthy populations were included. Studies on specific groups or non-healthy populations (e.g. children with a disability, cancer, clinical populations, clinically overweight/obese etc.) were excluded because these population groups often have unique PA patterns and related correlates that are specific for that population group and cannot be generalised to the wider population.

#### Dependent variable inclusion criteria

Studies needed to measure time-specific PA as a dependent variable, which was accrued during the school break time and after-school time periods. The school break time period was defined as any scheduled break time during school hours (e.g. lunchtime, morning/afternoon break). The after-school period was defined as the time between 3.00 – 6:00 pm (or within half an hour of the identified period), which is approximately the end of the school day until dinner time [41]. Studies investigating specific types of PA, such as active transport, sport and exercise, with no description of the time of day when these behaviours were performed, were excluded. Studies focusing solely on sedentary behaviour were also excluded because PA and sedentary behaviour are distinct behaviours with unique correlates [4,25,42]. It is acknowledged that sedentarism is an important behaviour but it is not the focus of this review. Recent reviews have been conducted on identifying the correlates of sedentary behaviour [24,43].

#### Independent variable inclusion criteria

The correlates measured in the studies needed to be tested for an association with PA that occurred either during the school break time or after-school periods. Studies that did not demonstrate this were excluded.

### Quality assessment of methodology

The methodological quality of cross-sectional studies and intervention studies that met the inclusion criteria were independently assessed by two reviewers (RMS, JD). Where there was disagreement between the two reviewers, consensus was reached by discussion. The quality of the cross-sectional studies was assessed using an eight-item quality assessment scale adapted from a previous review [44] for a school break time and after-school context. A score was assigned to each study based on whether the quality assessment items were sufficiently described (1), absent (0) or insufficiently described (?). The scores were summed and described as low quality (0–2), medium quality (3–5), and high quality (6–8). Intervention studies were assessed for quality using an 8-item assessment scale adapted from Van Sluijs et al. [8]. Each study was scored on an item based on whether it was sufficiently described (1), absent (0) or insufficiently described (?). The scores were summed and the quality classification was defined as low quality (0–2), medium quality (3–5) and high quality (6–8).

### Coding associations with physical activity

As no reviews of time-specific correlates have previously been conducted, all correlates measured in at least one study were included. Studies included in the review used a range of statistical techniques to evaluate the associations, including both univariate and multivariate analyses, which were adjusted for demographic and/or other potential correlates. Where possible, the adjusted model was used to evaluate the associations. Where results for gender and PA intensity were reported separately and different associations obtained, they have been treated as separate results and noted accordingly. If the associations for gender and PA intensity were reported in the same direction, the results were combined. This same approach has been used in a previous systematic review [45]. The correlates were grouped into six main categories of demographic/biological, psychological, behavioural, social/cultural, physical environment and policy. This categorisation of correlates were used by Sallis et al. [22] and subsequent reviews [19,24,25] and is based on a social ecological framework.

Correlates were coded based on statistical significance and the direction of association. The direction of the association between the correlate and time-specific PA was coded as either positive (+), inverse (−) or no association (0). No association was identified if there was a non-significant association between the independent variable and time-specific PA. The consistency of an identified association was determined by the number of findings supporting a hypothesised association. The cut-off coding was based on the codes used by Sallis et al. [22]. If 0-33% of the findings supported the association it was coded as “0”; if 34-59% of the findings supported the association it was defined as indeterminate and coded as “?”; and if 60-100% of the findings supported the association it was coded depending on the direction of the association, as either negative (−) or positive (+). Potential correlates related to school break time and after-school PA and the direction of association are reported separately in Tables 1 and 2. Correlates identified in intervention studies were reported separately as these indicate correlates of behaviour change [12]. These correlates can provide additional insights into which correlates should be specifically targeted to help promote time-specific PA and these are reported in Table 3.

**Table 1:** Summary of the associations of potential correlates with school break time physical activity across cross-sectional studies (n = 7)

**Correlate**	**Association**	**Reference**	**Summary (n**^**a**^**)**			
			**0**	**+**	**-**	**Assoc.**^**b**^
*Demographic/biological*						
Gender (males)	+	[50], [59-61], [62]VPA, [63]VPA	1	6	0	+
	0	[63]MPA				
Motor skills	+	[50]M	1	1	0	?
	0	[50]F				
Age	-	[59,61]	1	0	2	-
	0	[62]				
Family affluence (SES)	+	[61]	0	1	0	+
Body Mass Index (BMI)	0	[62]	1	0	0	0
*Social/cultural*						
Teacher supervision	+	[63]VPA	2	1	0	0
	0	[62], [63]MPA				
*Physical environment*						
Access to loose equipment	+	[62]MPA, [63]VPA M	2	2	1	?
	-	[63]VPA F				
	0	[62]VPA, [63]MPA				
Access to fixed equipment	+	[60], [63]MPA	2	2	1	?
	-	[63]VPA M				
	0	[59], [63]VPA F				
Playground markings	+	[63]MPA	2	1	0	0
	0	[62], [63]VPA				
Size of play space	+	[50]M	2	1	0	0
	0	[50]F, [62]				
Access to play space	+	[62]VPA	1	1	0	?
	0	[62]MPA				
Access to green space (no markings)	+	[60]	2	1	0	0
	0	[59], [63]				
Access to court space	0	[59,60]	2	0	0	0
Access to playing fields (with markings)	0	[59-61], [63]	4	0	0	0
Access to sledding hill	0	[59]	1	0	0	0
Access to ski tracks	0	[60]	1	0	0	0
Access to ice-skating areas	0	[60]	1	0	0	0
Access to fenced courtyard space	0	[60]	1	0	0	0
Access to climbing wall	0	[60]	1	0	0	0
Access to a wooded area	0	[60]	1	0	0	0
Access to water (sea, river, lake)	0	[60]	1	0	0	0
Access to bitumen areas	0	[63]	1	0	0	0
Access to outdoor obstacle course	+	[60]	1	1	0	?
	0	[59]				
Access to areas for hopscotch/skipping	0	[59]	1	0	0	0
Access to areas for board/skating	0	[59,60]	2	0	0	0
Access to indoor activity space	0	[61]	1	0	0	0
Access to a gym with cardio & weightlifting equipment	+	[60]	0	1	0	+
Access to swimming facilities	0	[60]	1	0	0	0
Number of facilities	+	[60]	1	1	0	?
	0	[59]				
Number of programs/activities	+	[61]	0	1	0	+
Access to facilities	+	[64]M	1	1	0	?
	0	[64]F				
Access to seating	0	[62]	1	0	0	0
Design of the school grounds	+	[64]F	1	1	0	?
	0	[64]M				
Condition of field	+	[61]	0	1	0	+
Condition of a gymnasium	0	[61]	1	0	0	0
Aesthetics	0	[64]	1	0	0	0
Length of recess time	0	[62]	1	0	0	0
Temperature	-	[62]VPA	1	0	1	?
	0	[62]MPA				
*Policy*						
PA school policy	0	[61]	1	0	0	0

Note: ^a^ n = the number of identified associations reported across studies (note: The number of studies are indicated in the reference column).

^b^Association shows the direction of the individual/summary association; + = positive association; - = negative association; 0 = no association or a non-significant association; ? = indeterminate.

MPA = moderate physical activity; VPA = vigorous physical activity; LPA = light physical activity; M = male; F = female.

**Table 2:** Summary of the associations of potential correlates with after-school physical activity across 10 studies

**Correlate**	**Association**	**Reference**	**Summary (n**^**a**^**)**			
			**0**	**+**	**-**	**Assoc.**^**b**^
*Demographic/biological*						
Gender (males)	+	[53-55]	1	3	0	+
	0	[57]				
Age	-	[52]F, [55]	1	0	2	-
	0	[51]F				
Ethnicity	-	[52]Hispanic or other F	2	0	1	0
	0	[52]African American White F, [55]				
Perception of general health	0	[54]	1	0	0	0
BMI	-	[51]F, [52]F	0	0	2	-
SES	0	[52]F	1	0	0	0
*Psychological*						
Self-efficacy (overcoming barriers)	+	[65]	1	1	0	?
	0	[55]				
Self-efficacy (support seeking)	+	[55]VPA	1	1	0	?
	0	[55]MPA				
Self-efficacy (competing activities)	0	[55]	1	0	0	0
PA enjoyment	+	[54]M	1	1	0	?
	0	[54]F				
Beliefs about PA	0	[55]	1	0	0	0
*Behavioural*						
TV viewing/playing video games	-	[51], [53]M, [55]	1	0	3	-
	0	[53]F				
Use of facilities	+	[52]F	1	1	0	?
	0	[58]				
Member of organised activities	0	[54]	1	0	0	0
*Social/cultural*						
Social influences	+	[65]	1	1	0	?
	0	[55]				
Peer support	+	[52]F, [54]M	2	2	0	?
	0	[51]F, [54]F				
Parent/family support	-	[54]F	2	0	1	0
	0	[52]F, [54]M				
Parent supervision	-	[29]F	0	0	1	-
Licence (parent influence)	0	[51]F	1	0	0	0
Perceived PA habits of parents/peers	0	[55]	1	0	0	0
*Physical environment*						
Access to facilities	+	[52]F, [54], [56]subjective F	2	3	0	+
	0	[51]F, [56]objective F				
Number of facilities	+	[56]subjective F,[58]subjective	3	2	1	0
	-	[57]F				
	0	[56]objective F, [57]M, [58]objective				
Number of amenities	0	[57]	1	0	0	0
Condition of facilities	0	[58]	1	0	0	0
Presence of walking & cycling paths	0	[57]	1	0	0	0
Presence of lighting along paths	0	[57]	1	0	0	0
Presence of trees	0	[57]	1	0	0	0
Presence of shade	0	[57]	1	0	0	0
Presence of a water feature	0	[57]	1	0	0	0
Presence of signage re dogs	0	[57]	1	0	0	0
Presence of signage restricting other activities	0	[57]	1	0	0	0
Park coverage	0	[58]	1	0	0	0
Land use mix	0	[58]	1	0	0	0
Access to equipment	+	[55]MPA	2	1	0	0
	0	[54], [55]VPA				
Neighbourhood safety	+	[54]	2	1	0	0
	0	[51]F, [58]				
Environmental barriers to AT	0	[54]	1	0	0	0

*Males are the reference point for gender associations.

^a^n = the number of identified associations reported across studies (note: The number of studies are indicated in the reference column).

^b^Association shows the direction of the individual/summary association; + = positive association; - = negative association; 0 = no association or a non-significant association; ? = indeterminate.

MPA = moderate physical activity; VPA = vigorous physical activity; M = male; F = female; objective = objective measurement of physical activity; subjective = subjective measurement of physical activity.

**Table 3:** Summary of the associations of potential correlates with school break time physical activity across intervention studies (n = 5)

**Correlate**	**Association**	**Reference**	**Summary (n**^**a**^**)**			
			**0**	**+**	**-**	**Assoc.**^**b**^
*Physical environment*						
Access to loose equipment	+	[46], [47]MPA VPA MVPA, [49]	0	3	1	+
	-	[47]LPA				
Access to fixed equipment	+	[49]	0	1	0	+
Playground markings	+	[46], [48,49]	0	3	0	+
Size of/access to play space	+	[46], [50]	0	2	0	+
Length of recess time	+	[49]	0	1	0	+

Note: ^a^ n = the number of identified associations reported across studies (note: The number of studies are indicated in the reference column).

^b^Association shows the direction of the individual/summary association; + = positive association; - = negative association; 0 = no association or a non-significant association; ? = indeterminate.

MPA = moderate physical activity; VPA = vigorous physical activity; LPA = light physical activity; MVPA = moderate to vigorous physical activity; M = male; F = female.

## Results

### Characteristics of the studies reviewed

**Figure 1:**
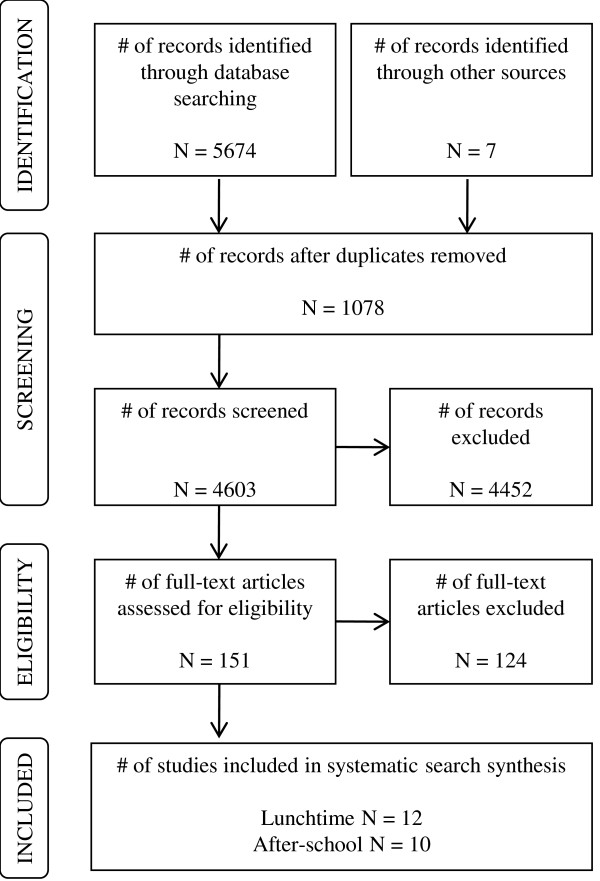
Flow chart of search results.

Of the 5681 studies identified from the electronic database and manual searches, 151 studies met the inclusion criteria, based on their titles and abstracts. After reviewing the full-text of these studies in more detail, only 22 studies met the inclusion criteria for this systematic review, 12 of which related to school break time PA and ten studies related to after-school PA. The main reasons for exclusion of some full-text articles were: the age of the sample, the study design, the study was a duplicate, the school setting was a secondary school, the focus was a specific behaviour without reference to a specific time period, the dependent variable was habitual PA (i.e. not time specific), the study did not measure potential correlates, and PA was not the dependent variable. Please refer to Figure 1, which demonstrates how the final number of studies was identified for inclusion in the systematic review.

Table 4 summarises the study characteristics, including the study design, theoretical framework and measurement methods. Of the studies included in the review, five were intervention studies, with two being randomised controlled trials [46,47], two being quasi-experimental studies [48,49] and one using a pretest-posttest design [50]. Nine studies focusing on the after-school period [29,51-58] and six studies focusing on school break times [50,59-63] were cross-sectional in design. One school break time study [64] and one after-school study [65] were questionnaire validation studies, containing correlational data. Studies were published between 1997 and 2010, with only two of these studies published prior to 2000, and conducted across a number of different countries, all of which are developed countries. Only five studies (two school break time [62,63] and three after-school studies [29,54,57]) based their research on an ecological theoretical framework, while the remaining were either based on behavioural theories, such as Social-Cognitive Theory [51,55,65] and Theory of Reasoned Action [55,65], or there was no discussion of the theory [46-50,52,53,56,58,61,64].

**Table 4:** Descriptive statistics of the studies included in the systematic review

	**Summary Statistics**	
	**School Break Time**	**After-School**
**Country**		
Australia	[50,63]	[54,57]
Norway	[59,60]	-
United Kingdom	[48,49,62,64]	[58]
United States	-	[29,51-53,55,56,65]
Canada	[61]	-
Belgium	[47]	-
Cyprus	[46]	-
**Study design**		
Observational (Cross-sectional)	[50,59-63]	[29,51-58]
Interventional (Randomised Controlled Trial)	[46,47]	-
Interventional (Pretest-Posttest)	[50]	-
Interventional (Quasi-experimental)	[48,49]	-
Questionnaire validation study	[64]	[65]
**Theoretical framework**		
Social Ecological model	[62,63]	[29,54,57]
Environmental correlates of an Ecological framework	[59,60]	-
Social Cognitive Theory	-	[51,55,65]
Theory of Reasoned Action	-	[55,65]
Not discussed	[46-50,61,64]	[52,53,56,58]
**Assessment of physical activity**		
Observation	[50,62,63]	-
Objective - accelerometry	[47,49,50,64]	[29,51-53,56,57]
Objective - heart rate	[48,49]	-
Objective - pedometer	[46]	-
Self-report	[59-61]	[29,54,55,58,65]
**Assessment of correlate variable**		
Observation	[46-50,62,63]	[51,52]
Proxy-report (school principal)	[59-61]	-
Proxy-report (parent)	-	[52,53,57,58]
Self-report	[59-62]	[29,51-56,65]
Objective	[62,64]	[52,56,58]

School break time PA was assessed using a range of methods, including observation (SOPLAY [50,63] and SOCARP [62]), objective measures (accelerometry [47,49,50,64], heart rate [48,49] and pedometry [46]), and self-report measures [59-61]. Two studies used self-report measures of PA but did not disclose the exact tool used [59,60]. After-school PA was only measured using accelerometry [29,51-53,56,57] and self-report (Previous Day Physical Activity Recall (PDPAR) [55,58,65] and 3 day Physical Activity Recall (3dPAR) [29]). One study [54] did not specify the self-report tool used for measuring after-school PA. The independent variables were predominantly assessed through self-report [29,51-56,59-62,65], observation [46-52,62,63] or proxy-report [52,53,57-61].

### Methodological quality of the studies reviewed

All the school break time studies using a cross-sectional design were assigned a methodological quality score of three or more, with one study considered high quality with a score of six out of eight [62]. Of the ten studies assessing correlates of after-school PA, eight were medium quality [51-54,56-58,65] and two were assessed as high quality (i.e. ≥6) [29,55]. Very few studies randomly selected the study sample [29,50,54]. Only three school break time studies and five after-school studies reported both a valid PA measure and correlate measure(s) with appropriate psychometric properties [29,50,51,55,57,62,64,65]. The remaining studies used either PA measures with poor or unknown validity/reliability [63], correlate measures with poor or unknown validity/reliability [53,56,58] or both [54,59-61]. No studies reported a power calculation; therefore, it was unclear whether they were adequately powered to detect hypothesised relationships between the PA behaviour and correlate. Four school break time studies [59-62] and seven after-school studies clearly described and accounted for potential confounders in analyses [29,52-56,58]. The majority of studies provided a clear description of the context of the specific PA behaviour (e.g. location in which the PA occurred, length of time engaged in PA).

The methodological quality of intervention studies ranged from medium [46-48,50] to high [49]. Key baseline characteristics for the intervention and control groups were adequately described and statistically tested in all studies. None of the studies clearly described the process of randomisation. Only four studies used PA measures that had been validated in the participant age group [46,47,49,50], with only one of these studies also using a correlate measure with reported psychometric properties [49]. Ridgers et al. [49] was the only study to use a six month follow-up, with the other studies using follow-up periods of four weeks [46] to four months [47,48]. Only two studies accounted for potential confounders in analyses [47,49]. No studies reported a power calculation to determine whether the sample size was adequate to detect hypothesised relationships.

### Correlates of school break time and after-school physical activity

Potential correlates of school break time and after-school PA were extracted and have been categorised separately according to the social ecological framework (demographic/biological, psychological, behavioural, social/cultural, physical environment and policy) (see Tables 1–2). Across the seven cross-sectional school break time studies, 39 potential correlates were identified, of which 20 correlates (56%) were investigated just once, ten (21%) were investigated twice, five correlates (13%) were investigated three times, and four (10%) were investigated four or more times (see Table 1). In the intervention studies, five correlates of PA change in school break time PA were investigated, with two correlates being investigated once, one correlate investigated twice and two correlates being investigated three or more times (see Table 3). Thirty-six potential correlates were identified in the cross-sectional after-school studies, with 20 correlates (56%) investigated once, six (17%) were investigated twice, five correlates (14%) were investigated three times and five (14%) were investigated four or more times (see Table 2).

#### Potential correlates of school break time physical activity (Tables 1 and 3)

Five demographic/biological correlates were examined across ten studies, including gender, age, family affluence, motor skills and body mass index. Gender was the most frequently studied correlate, with males being significantly more active than females during the school break time [50,59-63]. Age was explored in three studies [59,61,62], with two studies finding a negative association with school break time PA [59,61]. Motor skills, assessed in one study [50], were found to be important in male children but had no association with females’ school break time PA, resulting in an overall classification of indeterminate.

Teacher supervision was the only social/cultural correlate explored in the school break time setting [62,63]. No overall association was found between teacher supervision and school break time PA.

Thirty-two physical environmental correlates were examined in the school break time period, six of which were studied three or more times. Access to loose equipment [62,63] and fixed equipment [59,60,63] were the most frequently studied correlates and the association with school break time PA was inconclusive. Access to play space had a positive association with vigorous PA but no association with moderate PA during the school break time. A positive association was found for access to facilities [64], access to a gym [60] and condition of a playing field [61]. In one study, children were more active where there was greater provision of programs/activities [61]. Inconclusive evidence was found for overall facility provision (i.e. the sum of facilities available). Temperature was explored in one study [62], with evidence of an inverse association with vigorous PA during the school break time and no association found with moderate PA during school break time. Playground markings, size of play space, access to specific play spaces (e.g. court space, playing fields, sledding hill, bitumen areas), access to swimming facilities, access to seating, condition of a gymnasium, aesthetics and length of recess time were determined to have no association with school break time PA (see Table 1). Provision of a PA school policy was the only policy correlate explored [61]. No association was found between this correlate and school break time PA.

In the intervention studies, access to loose equipment and fixed equipment, playground markings, size of/access to play space and length of recess time were all manipulated to determine the effect on school break time PA. All five physical environmental correlates were found to positively facilitate change in school break time PA (see Table 3).

#### Potential correlates of after-school physical activity (Table 2)

Six demographic/biological correlates were addressed across seven different studies. These correlates included gender, age, ethnicity, perception of general health, body mass index (BMI) and socio-economic status (SES). There was evidence of a negative association between age and after-school PA [52,55]; and female BMI and after-school PA [51,52]. Gender had a positive association with after-school PA, with males being more active than females [53-55]. No associations were found for SES, perception of general health or ethnicity.

There was limited evidence of associations with psychological correlates, with only three studies investigating this domain. Self-efficacy in seeking support was positively associated with after-school vigorous PA, but not moderate after-school PA [55]. PA enjoyment was positively associated with after-school PA for males but not for females [54]. Evidence for an association between self-efficacy in overcoming barriers and after-school PA was inconclusive. No association was found for self-efficacy (competing activities) and beliefs about PA.

Behavioural correlates included TV viewing/playing video games, use of facilities and membership in organised activities. TV viewing/playing video games was the most frequently studied behavioural correlate, with evidence of a negative association with after-school PA [51,53,55]. The evidence for an association between after-school PA and the use of facilities was indeterminate, while no association was found for membership of organised activities.

Six social/cultural correlates were examined across six studies in the after-school period. Parent supervision was found to have a negative association with after-school PA for females [29]. Evidence for an association with social influences and peer support was inconclusive. No associations were found for parent/family support, licence (parent influence) and perceived PA habits of parents/peers.

Sixteen individual physical environmental correlates were identified, three of which were studied three or more times. Correlates included access to PA facilities (e.g. playgrounds, playing fields and dance studios), condition of facilities, presence of specific structures, access to equipment and neighbourhood safety. Access to facilities had a positive association with after-school PA. No association was found between the number of facilities and after-school PA. However, when viewed more closely, there was a consistent positive association between the number of facilities and after-school PA when the number of facilities was subjectively assessed [56,58]. No associations were found for neighbourhood safety, environmental barriers to active transport, access to equipment, land use mix, park coverage, presence of specific structures, condition of facilities and number of amenities.

## Discussion

This review has provided an overview of the current evidence and quality of evidence for influences on children’s PA behaviour during two periods of the day, the school break time and after-school time period. Despite these day segments being identified as “critical” periods for PA promotion [25,41], they remain relatively unexplored. Past reviews have identified correlates of whole day PA behaviour for children and adolescents but have not explored correlates relating to a specific time, location or behaviour context [19,22,24]. To date, no other review has been conducted on the correlates of time-specific PA in children from a multi-factorial perspective. Ferreira et al. [25] focused on environmental correlates in location-specific settings (i.e. home, school and neighbourhood) but did not examine other domains of the ecological model, limiting our understanding of the multi-dimensionality of setting-specific PA behaviour.

Relatively few studies met the inclusion criteria for this review, with the majority of included studies only exhibiting medium methodological quality, highlighting the seriously limited evidence upon which setting- and context-specific PA interventions can be based. Due to the paucity of high quality evidence, the findings of this review cannot be used to draw definitive, meaningful conclusions about the correlates of time-specific physical activity and should be interpreted with some caution. Age and gender, with boys and younger children more active, were consistently associated with school break time PA in two or more studies [50,59-63]. Family affluence [61], access to a gym [60], access to four or more PA programs [61] and the condition of a playing field [61] were all associated with school break time PA in one study. Access to loose and fixed equipment [46,47,49], playground markings [46,48,49], size of and access to play space [46,50] and the length of school break time [49] were all positively associated with changes in school break time PA in intervention studies. In the after-school period, gender (with boys again more active) [53-55], younger age [52,55], lower body mass index (for females) [51,52], lower TV viewing/playing video games [51,53,55] and greater access to facilities [52,54,56] were associated with higher levels of after-school PA in two or more studies, while parent supervision was negatively associated with females’ after-school PA in one study [29].

Higher levels of PA among boys compared with girls are consistently reported in the literature, regardless of whether comparisons relate to total PA [22,24,45] or context-specific PA, as shown in this review. These differences may be explained by underlying biological mechanisms but may also be attributable to the social context of the specific time-period. Boys typically view school as a chance to engage in competitive games that tend to dominate play spaces in the school yard, while girls view the school break period as a time for socialising [66]. During the after-school period, evidence suggest that parents perceive the neighbourhood to be safer for adolescent boys compared to adolescent girls [67], which may contribute to the observed gender differences in this time period.

Recent reviews present an inconsistent picture in relation to age and PA among children and adolescents. Sallis et al. [22] found a consistent negative association with habitual PA among adolescents, while Van der Horst et al. [24] reported inconclusive evidence in adolescents. In the current review, age was found to be negatively associated with both the school break time and after-school periods. However, it should be noted that this was only assessed in three studies. Consequently, there is a need for additional research into the relationship between age and time-specific PA. As gender and age are non-modifiable correlates of PA, the development of effective interventions to promote PA will depend on a deeper understanding of how these biological attributes interact with the settings and contexts within which PA occurs [68]. This requires further research into environmental factors specific to different periods of the day.

The current review identified a negative association between TV viewing and after-school PA. Hohepa et al. [69] also found a significant inverse association of TV time with after-school PA among adolescents, suggesting that TV viewing is in direct competition with PA opportunities in this time period. Notably, other correlate reviews [22,24] found no association of screen time with habitual activity. While TV viewing may not influence whole day PA, these findings, along with results from Hohepa et al. [69], provide some initial support that TV viewing may negatively influence PA during periods of relatively high TV accessibility. Recent evidence has shown that TV viewing is the most prevalent activity performed by children of this age group during the after-school period [41,70], and could potentially be an important intervention target as a means of increasing after-school PA.

In the present study, ‘access to facilities’, represented as a composite variable, was correlated with after-school PA, with inconclusive evidence in relation to school break time PA. Previous reviews have tended to collapse specific facility correlates into a general ‘access to facilities’ score, which may mask important context-specific associations. Using this approach, Sallis et al. [22] found a consistent association between access to facilities and habitual PA, whereas the findings from Ferreira et al. [25] and Van der Horst et al. [24] did not support this association. Access to specific facilities, such as swimming pools and playing fields, was examined separately in this review in order to minimise loss of contextual information, however, these specific facility correlates were represented too infrequently to draw any conclusions. Future studies using a context specific approach should identify and report specific facilities relevant to the context in question, which will contribute to a clearer understanding of context-specific PA.

The relatively small number of studies that met inclusion criteria for this review varied in terms of theoretical framework, study design, sample characteristics, measurement techniques, analytical approaches, and representations of PA. Thirteen studies used objective PA measures while eight studies used self-reported PA measures. Twelve studies used an objective correlate measure while 14 studies used a self-reported correlate measure. Only eight studies reported relevant psychometric properties of both the PA and correlate measures. Associations between PA and a potential correlate have been shown to differ depending on how PA or the correlate was measured [25,71]. Future studies should choose measurement tools with appropriate psychometric properties. Analytical techniques varied across studies with some using univariate [29,47,50,51,53,57,63-65] and others using multivariate strategies [46,48,49,52,55,58,61,62]. Multivariate analysis can result in fewer significant associations [24], and the order in which correlates are entered into multivariate models can influence final model structure. Therefore, differences in analytical approaches among studies are likely contributors to the current confusion in the literature [12].

The current review identified a small number of studies that varied widely in important methodological aspects, thereby limiting the generalizable conclusions that can be drawn. Further, there are limitations of the review process that need to be acknowledged. Firstly, there may have been studies that were missed because of the search terms used, or unclear titles or abstracts. Secondly, due to the relatively high proportion of cross-sectional studies included in the review, it is not possible to identify those correlates of PA behaviour change that would provide the most powerful evidence for intervention design. Some studies stratified analyses by salient variables such as age, gender and intensity of PA, resulting in an over-representation of these studies in the review. While this level of specificity is important in correlate research, there may be consequent bias towards studies that reported numerous associations compared to those that reported few associations. Finally, the relatively narrow age range specified in the current review is a limitation. We do acknowledge that the age at which children transition from primary to secondary school may differ internationally and factors influencing PA of 8 year olds and 14 year olds may differ [39,40]. To minimise the effect of this on the review’s findings, only studies conducted in primary schools were included. Summarising the findings for narrower age ranges (i.e. 8–12 and 13–14 year olds) is not feasible due to the small number of studies in this area.

## Conclusions

While there is strong evidence that school breaks and after-school periods are ‘critical windows’ for PA promotion among young people, this review has clearly identified the paucity of high quality evidence upon which PA promotion in young people can be tailored to specific settings and contexts. The relatively small number of studies provided preliminary evidence that the intra-personal and inter-personal influences on PA vary according to different contexts such as the school break time and after-school periods. However, the review also exposed a lack of clarity in this area and underscores the importance of focusing attention on context- and setting-specific PA among young people.

## Competing interests

The authors declare that they have no competing interests.

## Authors’ contributions

RS contributed to the conceptualisation and design of the manuscript, conducted the literature review, collected and analysed data, and drafted the manuscript; KR and JD contributed to the conceptualisation of the manuscript, data extraction, and provided substantive feedback on the manuscript. All authors read and approved the final manuscript.

## Funding disclosure

No funding to disclose.
